# Machine Learning Prediction of Allosteric Drug Activity from Molecular Dynamics

**DOI:** 10.1021/acs.jpclett.1c00045

**Published:** 2021-04-12

**Authors:** Filippo Marchetti, Elisabetta Moroni, Alessandro Pandini, Giorgio Colombo

**Affiliations:** † Department of Chemistry, Università Degli Studi di Pavia, Viale Taramelli 12, 27100 Pavia, Italy; ‡ Università Degli Studi di Milano, Via C. Golgi, 19, I-20133 Milan, Italy; § Istituto di Scienze e Tecnologie Chimiche, Via Mario Bianco 9, 20131 Milano, Italy; ∥ Brunel University London, Uxbridge UB8 3PH, U.K.

## Abstract

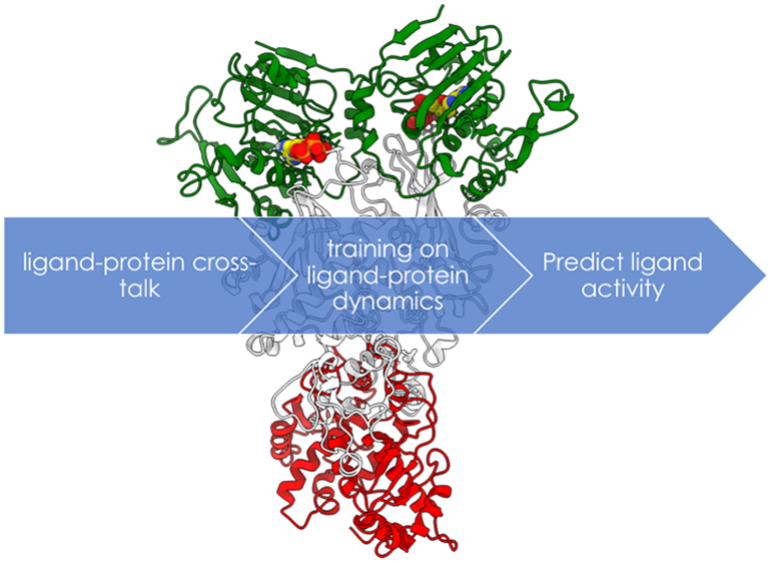

Allosteric drugs have been attracting increasing interest over the past few years. In this context, it is common practice to use high-throughput screening for the discovery of non-natural allosteric drugs. While the discovery stage is supported by a growing amount of biological information and increasing computing power, major challenges still remain in selecting allosteric ligands and predicting their effect on the target protein’s function. Indeed, allosteric compounds can act both as inhibitors and activators of biological responses. Computational approaches to the problem have focused on variations on the theme of molecular docking coupled to molecular dynamics with the aim of recovering information on the (long-range) modulation typical of allosteric proteins.

Here, we present a protocol that combines docking-based screening, information on the conformational dynamics of the protein, and machine learning (ML) to classify ligands of the molecular chaperon Hsp90 as activators or inhibitors. To this end, we develop a classifier of activation/inhibition of Hsp90 allosteric ligands that is trained on data from a panel of ensemble docking results. The data set for this study is built from a database of 133 known Hsp90 ligands.

Three different ML methods are compared with the best-performing algorithm, achieving an average balanced accuracy of 0.90 (over 10-fold cross-validation) in correctly separating inhibitors from activators. A comparison with a direct classification of the chemical properties of ligands suggests that the ML prediction is not dependent on the similarity among the molecular structures but recovers hidden similarities in functional effects of different ligands.

The improved knowledge of gene organization coupled with the advances in gene editing and structural analysis methods can potentially start a whole new era in drug discovery.^1,2^ In particular, improved target identification can shed light on biomolecules whose perturbation via small-molecule binding results in a functional response, transforming a disease phenotype into a normal one. The extraordinary complexity of biochemical networks in healthy and disease conditions^3,4^ and the costs associated with drug discovery are however hampering the advent of this new era of therapeutics, as shown by the relatively low numbers of new drugs approved in the past few years.^5,6^ Most drug discovery efforts aim at targeting the active sites of enzymes or the orthosteric sites of regulatory proteins. Because of the evolutionary and structural conservation of such sites across the proteome, issues related to selectivity, off-target effects, and development of drug resistance have started to appear.

In this context, allosteric ligands have recently emerged as a viable complement or alternative to active-site directed molecules, with novel potential as drug candidates or chemical tools.^7−10^ Allosteric ligands bind to sites that are generally distinct and distal from the classic orthosteric ones. In doing so, they can perturb the target not only by inhibition but also through modulation or activation of specific functions. This represents an advantage in terms of fundamental and applicative perspectives. In fundamental research, chemical modulators (effectors) can be used to direct signaling pathways and whole cells toward desired functional states, representing important tools for understanding the roles of specific biomolecules in complex biochemical networks.^11,12^ In biomedical applications, since they target sites that are generally less evolutionarily conserved, allosteric ligands can be highly selective, even among different members of the same protein family,^13^ providing new opportunities for therapeutic discovery.

To date, most (non-natural) allosteric ligands/drugs have been discovered using high-throughput screening. The ever growing amount of sequences and structural information combined with the increases in computing power and the improvement of predictive algorithms are starting to facilitate the discovery of allosteric modulators, but major challenges remain to develop approaches focused on rational drug design.

Computational approaches to the problem have focused on variations on the theme of molecular docking. Binding affinities predicted by docking simulations are routinely used in virtual screening to estimate relative ligand rankings and to inform further steps in lead identification.^14,15^ Efficient screening of large libraries of compounds is achieved by the use of approximate scoring functions and simplified strategies for conformational sampling.^16^ Typically, a static model of the target structure is used. However, recently the influence of protein dynamics on the recognition process has been more accurately modeled using ensemble strategies.^17−22^ These strategies involve the docking of a molecular ligand libraries over an ensemble of selected geometries of the protein, creating a more realistic representation of the ligand bound to the different expected conformations of the target. The use of an ensemble of conformations reduces the dependence of the docking results on the target structure.^23^ Ensembles can be extracted from unbiased molecular simulations of apo structures^24^ and more often by sampling of protein conformations from holo structures containing first-generation ligands.^25^ Under the assumption of conformational selection, a set of different ensembles representing different binding states would have selective preferential binding for different ligands. On the basis of this hypothesis, previous studies have used “a panel of ensembles” for virtual screening,^26^ whereby a vector of binding affinities against the panel is used to generate a specific fingerprint for each ligand.

This type of data has high dimensionality both in the chemical and conformational space and is best suited for analysis using ML methods, which have been increasingly adopted in drug discovery studies. Indeed, they contributed to the improvement of performance in virtual screening studies^27−29^ and they have been effectively used in the enhancement of structural-based virtual screening and scoring.^30,31^ ML methods are mostly data-driven, and their performance is often dependent on the size and quality of the data set. To this end, they may present limited transferability, and care is required in reporting results and the scope of applicability.

The combination of ML with molecular simulations can dramatically advance the process of selection of allosteric ligands with a desired impact on the function of the target.^32^ Indeed, a major limitation in docking simulations is the lack of information on the functional consequences of the allosteric binding event. While relative binding affinities and geometries can be reproduced close to experimental accuracy, there is no predictive score to discriminate inhibitors from activators, agonists from antagonists or partial agonists.^33^ Experimental assays typically report on the orthosteric function, in most cases by direct measurement of a relevant biochemical parameter that involves the active/orthosteric site. This may not necessarily reflect the affinity of binding at the allosteric site.^34−36^ In most cases, binding is only one aspect of an intricate interplay of structural and dynamic factors that emerges from the cross-talk between the allosteric ligand and the protein and define functional responses. As a consequence, the derivation of structure–activity relationships (SARs) for allosteric ligands is typically much more complex than for orthosteric ones.

This unmet challenge calls for new approaches that integrate information on binding, conformational dynamics, and biological activity because the desired readout of the binding event is a change of functional state in the protein that is not directly or easily modeled by single docking calculations.

Here, to progress along this fascinating avenue, we explore the potential of ML models trained on molecular simulations to predict the functional effect of allosteric ligands on proteins. Allosteric ligands can either activate or inhibit protein function. As a test case, we focus on the difficult case represented by the Hsp90 chaperone system, a molecular machinery essential for cell development and maintenance that works by facilitating the folding of a broad spectrum of clients.^37−42^ Proteins of the Hsp90 family (Hsp90 in the cytosol, Grp94 in the ER and Trap1 in mitochondria) are homodimers with two chains consisting of three globular domains, the N-terminal (NTD), middle (M), and C-terminal (CTD). The functions of the chaperone are regulated by ATP hydrolysis in the NTD, where ATP processing is coupled to Hsp90 conformational reorganization and consequent client remodelling (see Figure 1). Early work by Neckers’ group and recent computational studies reported an allosteric site at the boundary between the M- and C-terminal domains that modulates ATP-related functionalities^10,43−45^ (see Figure 1). The discovery of this allosteric site facilitated the development of different series of allosteric ligands that are able to perturb Hsp90 mechanisms, by either inhibition or activation of ATP processing. Kinetic and biochemical data indicated that the functional effects of the ligands are critically coupled to their influence on the conformational dynamics of the protein.

**Figure 1:**
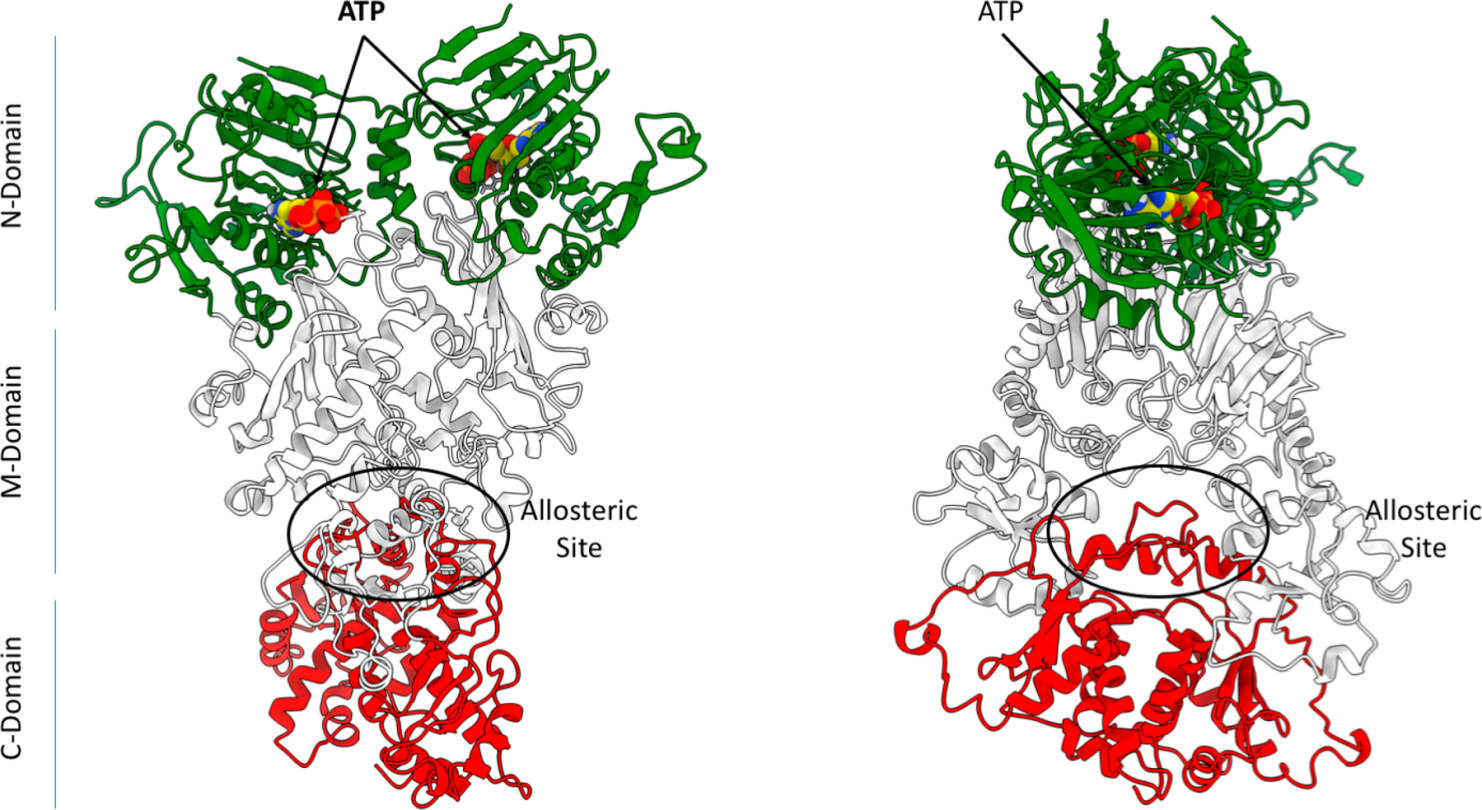
3D structure and domain organization in Hsp90. The N-domain is colored in green, the middle in white ,and the C-domain in red. The ATP molecule is shown in its binding site in van der Waals representations with atom type coloring. The allosteric site is also highlighted.

In this work, we ask whether we can develop a reliable predictor of activation/inhibition for Hsp90 allosteric ligands. Model training is driven by ensemble-based structural, dynamic, and energetic characterization of allosteric binding.

Our approach to classify allosteric ligands as activators or inhibitors of ATP hydrolysis in Hsp90 entails three steps (see Figure 2).

**Figure 2:**
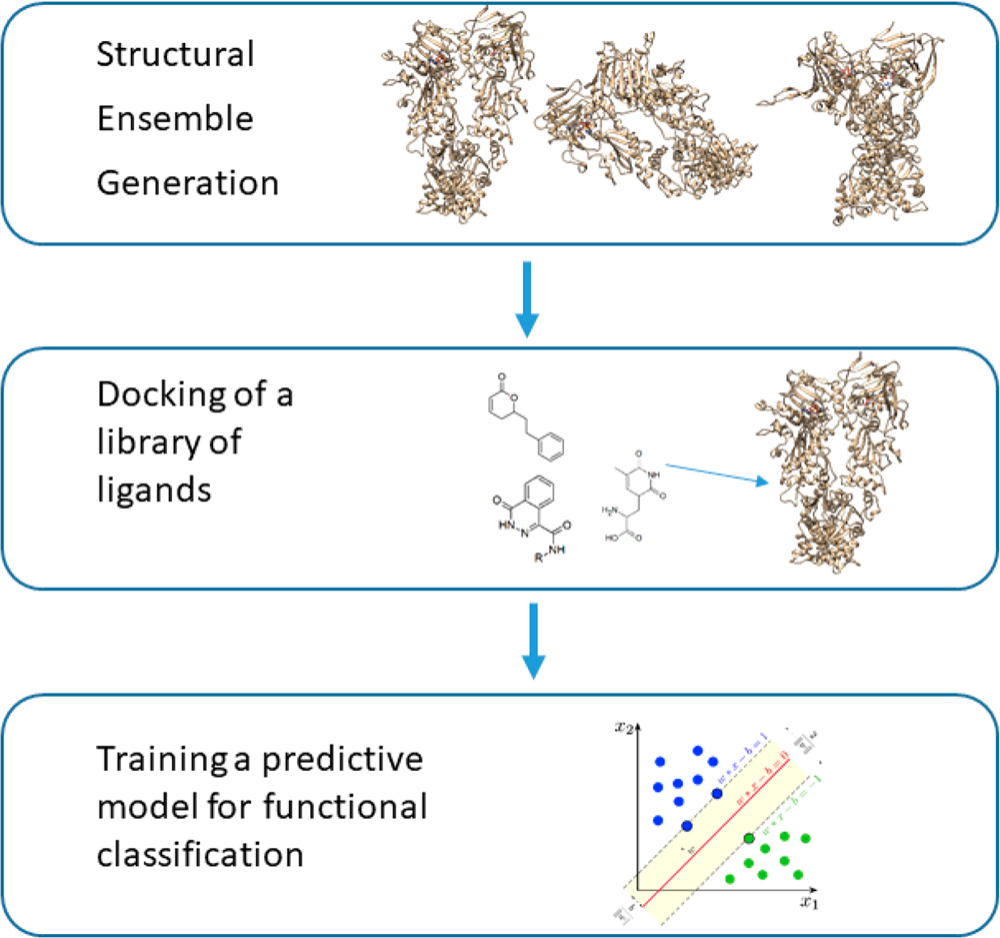
Simplified scheme of the MD-ML strategy. Schematic representation of the protocol followed in this work, which entails the generation of a structural ensemble, the docking of ligand libraries, and the training of learning algorithms for functional classification.

First, a panel of structural ensembles is generated by cluster analysis of conformations from molecular dynamics simulations of representative holo structures, in which Hsp90 is bound to ATP in the N-terminal domain and to an allosteric effector in the allosteric site. Then a library of allosteric compounds is docked against the Hsp90 structural panel. Finally, a predictive model for functional classification of the allosteric ligands is trained taking into account the structural, dynamic, and energetic properties of the resulting complexes. From the literature, we collected 133 compounds with known activity against Hsp90, comprising 49 inhibitors and 84 activators (see supplementary Figure S1). This data set was used to train and test the predictive model. The protein conformational ensembles for docking were generated by atomistic molecular dynamics simulations in explicit water of Hsp90 in complex with three different ligands: one activator (CC26) and two inhibitors (ND2 and Novobiocin) (see supplementary Figure S2).

We note here that in our model, the dynamics of the protein and potential allosteric effects determined by the cross-talk between the ligand and the chaperone is taken into account explicitly in these preliminary simulations with the three representative of activators and inhibitors. In this context, it is worth noting that our work aims to investigate how short time-scale changes in the structural dynamics of the chaperone dimer in the presence of small molecule effectors may determine the onset of the motions that are eventually relevant for function. The underlying hypothesis is that nanosecond time scale residue fluctuations of Hsp90 in regions that are specifically responsive to the presence of ligands may facilitate the large-scale domain rearrangements that lead to a functionally competent/incompetent state. These concepts were previously probed via computational and experimental approaches.^46^

To keep the generation of the structural ensembles independent from the data set used for training, these ligands were not included in the training and test data sets.

Each replica of molecular dynamics was run for 400 ns, saving structures every 100 ps, and the resulting trajectories were combined into a single metatrajectory. The panel of structural ensembles for docking was built to take into account the conformational variations induced by the ligands and approximate the most relevant states in Hsp90 functionally oriented dynamics. To this end and to qualitatively account for the cross-talk between the presence of a ligand and the different domains of Hsp90, geometrical cluster analysis of the metatrajectory was repeated using four different reference frameworks: the backbone atoms of N-terminal domain (Clust-N); the backbone atoms of middle domain (Clust-M); the backbone atoms of N-terminal and middle domains (Clust-NM), and the backbone atoms of middle and C-terminal domains (Clust-MC). In addition to these domain-based frameworks, a cluster analysis of the allosteric site was performed, where the ligand binding site was defined as the ensemble of residues that are within 1 nm of any bound allosteric ligand in at least 75% of all visited structures collected in the metatrajectory. This latter criterion was used to consider the most relevant local interactions between residues in and around the binding pocket and the ligand. It is worth underlining here that, during MD, the spectrum of contacts dynamically evolves.

Next, the representative structures from the three most populated clusters for each of the four domain-based ensembles were selected as a target for docking experiments. The total number of structures in the three most populated clusters always account for at least 45% of the metatrajectory. In addition, the two main representative structures resulting from the allosteric-site based clustering were added. Two structures were enough to recapitulate more than 95% of the structural variability observed in the pocket.

Cluster analysis of the molecular dynamics metatrajectory yielded a total of 14 representative protein structures for the following step of docking. This collection was generated to capture the propensity of Hsp90 to populate conformations potentially endowed with different functional properties. After docking the ligand library to each of the selected representative structures, three measures were calculated for every resulting complex: the docking score of the best pose for every representative structure; the root-mean-square (RMS) of the docking score for the 10 best poses; and finally, the RMSD on the atomic positions of the first 10 poses, reporting on the structural adaptation within the pocket. Ten poses per ligand were selected as a compromise to provide a tractable and easy to visualize number of configurations, while capturing positional adaptation of the molecules to a changing binding site. A total of 42 features was thus used for ML prediction.

The underlining hypothesis of our study is that features describing the docking results of a ligand against a panel of distinct conformational ensembles can be used as “dynamic fingerprints” of its functional effect on the protein. We tested this hypothesis under three assumptions: (1) the separation of activators and inhibitors cannot be directly detected in the feature space by cluster analysis; (2) the separation of activators and inhibitors requires modeling a complex relationship by supervised learning; and (3) the separation cannot be trivially obtained by use of small molecule fingerprints in the absence of information on the protein structure and dynamics.

None of the features described above can independently be used as a classifier and directly separate inhibitors from activators. This is evident from the distribution of values for every single feature against the two known ligand classes: in all the cases, the pair of per-class distributions overlap (see boxplot in Supplementary Figure 3). This suggests that a model based on the combination of these features is required to discriminate between the two classes. The first step is to test if the separation of the two groups of ligands can be directly detected with an unsupervised learning approach.

To this end, cluster analysis was performed to assess if a data segmentation compatible with the two functional classes of ligands (activators/inhibitors) can be detected. Two different algorithms were used: k-means and agglomerative hierarchical clustering. The target cluster numbers could be set to 2, but we adopted an unbiased approach and explored values between 2 and 6. The ability to correctly separate ligand classes in the clusters was estimated by cluster purity, which has values between 0 (when the class labels are completely mixed in the clusters) and 1 (clusters composed by only one class). Both algorithms have similar purity values; in particular, when 2 clusters are considered, the purity is low (0.66 for K-means and 0.69 for hierarchical), and with more clusters, the purity increases, remaining below 0.80 (for 4 clusters: k-means have 0.78 of purity and hierarchical have 0.79). The increased purity is due to the reduced size of clusters that helps adapt to the class separation. Yet, the value in the case of 2 clusters reveals that is difficult to detect a segmentation of the compounds in the functional classes directly by cluster analysis. This suggest that it is not possible to automatically partition the space of the data to identify inhibitors and activators. A model trained on properties from the different binding conformations is therefore needed.

In this framework, a classification model was built using supervised learning. The model is trained to predict class labels describing the functional effect of the ligand (i.e., activation or inhibition). Three widely used algorithms were compared: Logistic Regression (LR) as a baseline, Support Vector Machine (SVM), and Random Forest (RF). The performances of the three methods was compared after training and testing using the holdout method, where the data set is randomly split in training set and test set with the proportion of 70% and 30% respectively. The performance in prediction is reported in Table 1.

**Table 1:** Performance of ML Approaches: Values of Balanced Accuracy, Precision and Recall, False Positive Rate, and False Negative Rate for All Three ML Models Tested in the Paper

measure	LR	SVM	RF
balanced accuracy	0.88	0.89	0.74
precision (positive predictive value)	0.92	0.96	0.81
recall (true positive rate)	0.88	0.85	0.85
false positive rate	0.12	0.07	0.37
false negative rate	0.12	0.15	0.15

LR and SVM show similar performances while RF has poorer performance. Nevertheless, all three methods show a better classification power compared with the cluster segmentation. A 10-fold cross-validation without shuffling was performed to exclude any bias due to the simple holdout split and to further compare the methods. This approach also highlighted possible variability across the data sets and facilitated interpreting the performance with more insight on the chemical features of the molecules (see below).

SVM shows the best performance with an average balanced accuracy of 0.90, compared with 0.87 (LR) and 0.79 (RF). In Figure 3, per-fold balanced accuracy is reported. Only for one fold, values are below 0.8. The results show consistency in performance by SVM across the set. To confirm that the model is properly trained, its convergence was assessed at the increase of the training set. For each subset size ranging from 20 to 100%, 100 random samples were generated. SVM models were trained and tested with holdout. Performance was evaluated as median accuracy over the 100 random samples. The accuracy converged to 0.89 for the subset at 80%. This suggests that with a data set of ∼100 compounds the model can be built with confidence (see Supplementary Figure 4).

**Figure 3:**
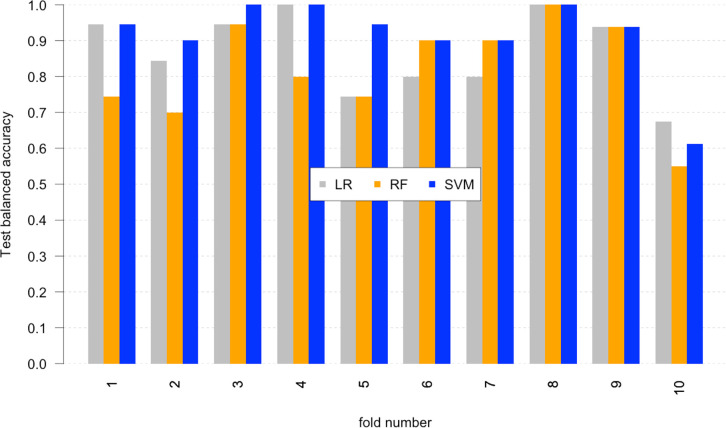
Performances of the 10-fold cross-validation for all the models. The values of balanced accuracy for every fold presented: the values for Logistic Regression are in gray, and Random Forest are in orange and SVM in blue.

Finally, the possible dominance of one type of ensemble feature (docking score, rmsd, rms) in the prediction was assessed by selectively excluding each feature in turn and repeated cross-validation. In each case the variation in the average performance was not statically significant (i.e., z test score below 1; see Supplementary Figure 5), and therefore, no feature was detected as dominant.

The classification model trained on docking against the panel of representative conformations does not directly account of chemometrics properties of the ligands. In the context of compound selection, it is interesting to compare the classification model with a direct analysis of the chemical properties of the compounds. This is to assess if correct classification can be obtained by small molecule fingerprints in the absence of information on protein structure or dynamics.

Our data set comprises molecules representative of different chemotypes (Supporting Figure 1). It may be possible to qualitatively cluster these molecules with respect to shared scaffolds: in our case, this results in eight different groups (Figure 4).

**Figure 4:**
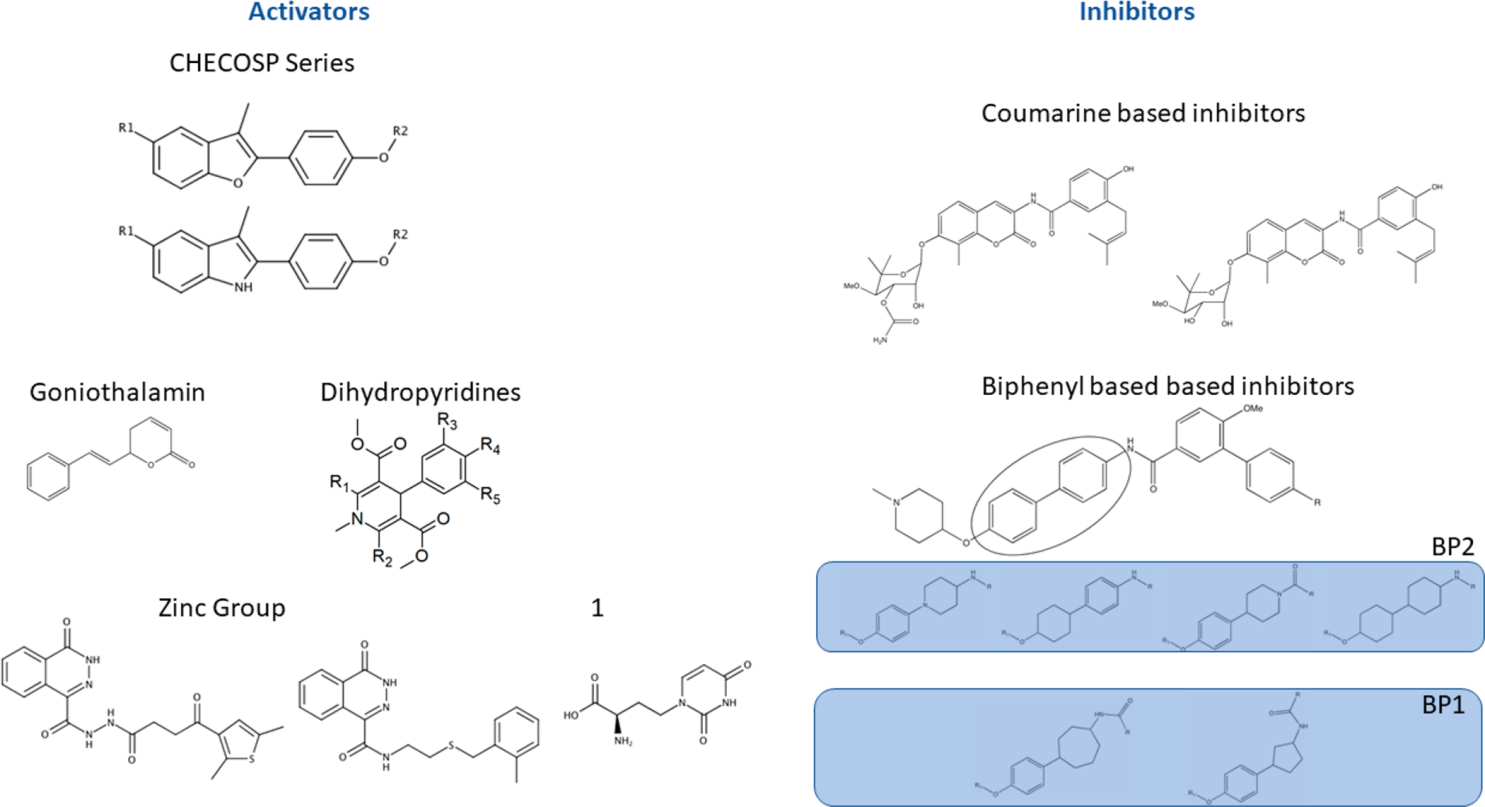
Subdivision of the studied molecules in distinct groups. The 2D structure of the scaffolds are divided in eight groups according to a scaffold-similarity criterion. From left to right: CheCOSP molecules (CC), coumarine-based inhibitors (CB), goniothalamin (GT), dihydropyridines (DP), the biphenyl inhibitors set is split in two groups (BP1 and BP2), the Zinc Group (Z), and last the compound labeled with 1 makes his own group (Unk).

Yet, the compounds can still display substantial differences in their substituents in terms of dimensions, charges, and functional groups. Therefore, a classification based only on the core of the molecules would give only a rough estimate of the chemical variability in the data set. For this reason, to explore the possibility of classifying the function of molecules based only on their chemical properties, we used a more quantitative method based on cheminformatics similarity criteria. A common method to evaluate the similarity among compounds is to compute the Tanimoto coefficient on molecular fingerprints.^47^ The efficacy of similarity algorithms tends to vary with biological activity; therefore, the choice of the fingerprint model usually depends on the system under study. Here, our aim is specifically to introduce a metric for the comparison with our ML-dynamics based predictions. Since the best fingerprint model for our data set is not known, we tried two widely used methods: ECFP, a method that maps a molecule with a set of fragments radially grown from each heavy atom; and MACCS, which accounts for the presence/absence of specific structural features.^48^ In both cases, the molecules are clustered using k-means algorithm with a cluster number varying from 2 to 6 (Table 2). The ECFP fingerprint works better in separating the compounds between activators and inhibitors when only 2 clusters are chosen, while with 3 or 4 clusters, the separation is similar. With 3 clusters, we found that the CheCOSP^20^ group is separated. This consists only of activators validated by experimental characterization. The result shows that, despite a shared scaffold, there is a substantial chemical variety in the group. Segmentation for higher cluster numbers does not clearly lead to any grouping consistent with the chemical properties of the ligands.

**Table 2:** Performance of the Fingerprinting Methods

	MACCS		ECFP	
	activator	inhibitor	activator	inhibitor
K = 2				
cluster 1	33	48	12	49
cluster 2	51	1	72	0
K = 3				
cluster 1	45	1	67	0
cluster 2	0	47	17	2
cluster 3	39	1	0	47
K = 4				
cluster 1	16	0	42	0
cluster 2	0	47	14	2
cluster 3	36	1	0	47
cluster 4	32	1	28	0
K = 5				
cluster 1	44	0	31	0
cluster 2	1	32	0	42
cluster 3	0	29	0	28
cluster 4	0	13	16	0
cluster 5	4	10	2	14
K = 6				
cluster 1	44	0	2	14
cluster 2	1	32	0	42
cluster 3	0	29	16	0
cluster 4	0	12	31	0
cluster 5	3	6	0	14
cluster 6	1	5	0	14

In Table 2, we report the performances of MACCS and ECFP fingerprints in recognizing and assigning activators and inhibitors, obtained with k-means clustering.

The best result obtained by ECFP fingerprint on two clusters was compared with the best ML predictive model obtained by SVM (all data in Supporting Table 1). The comparison was broken down by chemical groups to explore how the two approaches perform on different subclasses of ligands. In Figure 5, we report the fraction of correct classifications for every group in our data set. For the group of three inhibitors (BP1, BP2, and CB), a high fraction of correctly classified is observed for ECFP, meaning that inhibitors have good chemical similarity, whereas for activators, the fraction for ECFP is high only for CC group. In all the other groups (Z, DP, Unk, and GT), the fraction is 0. Interestingly, the SVM model correctly predicts as activators even the groups with low similarity with CC (the group most extensively characterized at the experimental level). In this context, we notice that SVM still correctly predicts group Z to 0.3 (0.0 in the case of fingerprints), DP to 0.6, Unk and GT to 1. In contrast, inhibitors of the CB groups have good similarity with the rest of the inhibitors, but they are not correctly predicted by SVM.

**Figure 5:**
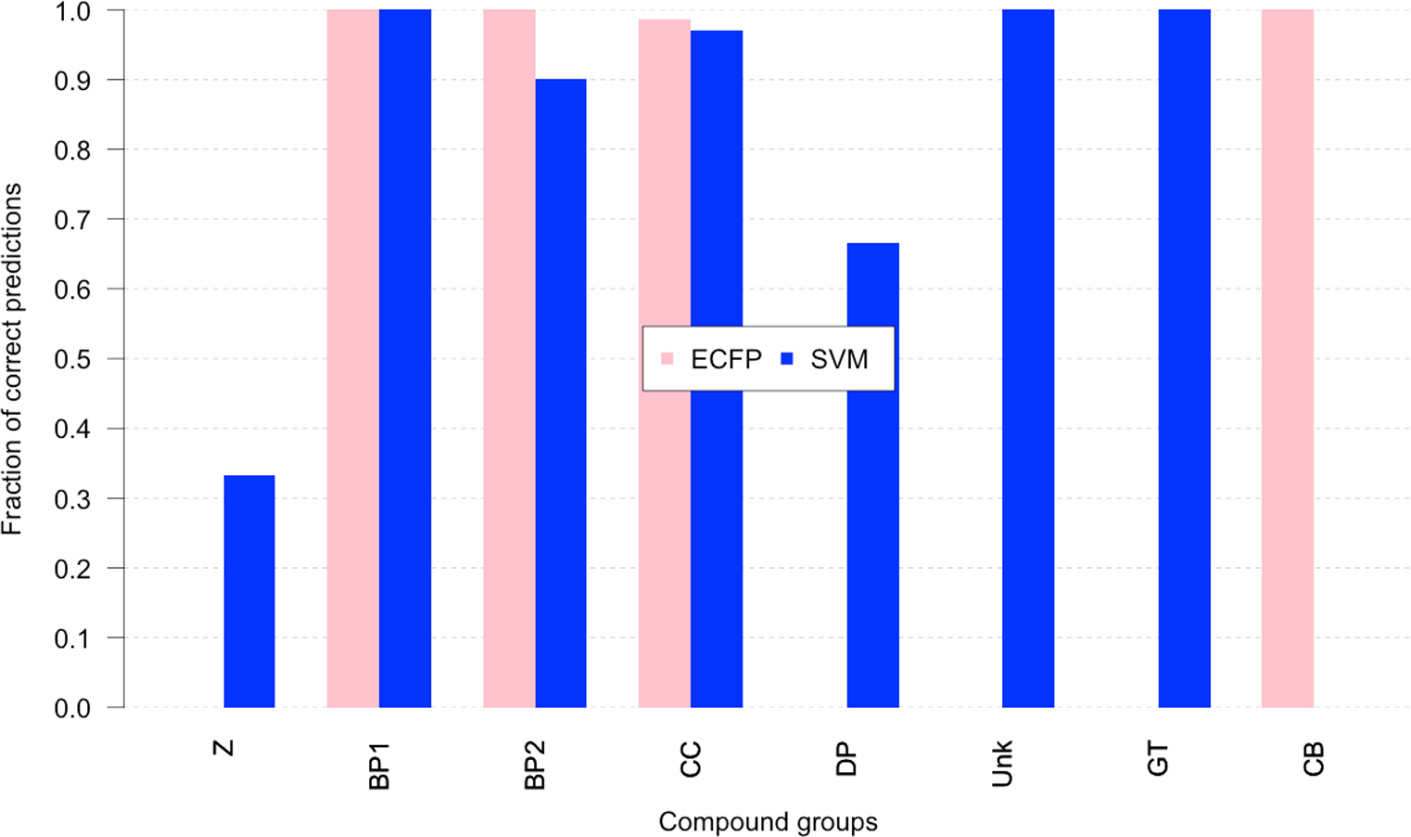
Performance comparison between SVM and ECFP. The graph reports the fraction of correct predictions obtained with the SVM method compared with the cluster separation of ECFP values. An entry of the ECFP cluster is considered correctly separated if it is located in the cluster that contains the majority of its class. The values are in pink for ECFP and in blue for SVM, the fractions are evaluated separately for every scaffold group

Overall, the results of this comparative analysis suggest that the characterization of allosteric binding with the partner protein, which reverberates the cross-talk between the ligand and the receptor, captures the main structural and dynamic determinants at the basis of allosteric modulation. On the one hand, this approach is not dependent on the similarities among the molecular structures of the libraries of compounds under exam. On the other hand, considering that specific functionalities may determine recognition, binding, and the successive functional regulation, it is important to underline that the relevance of specific chemotypes for functional modulation emerges from the ML analysis. This aspect is aptly captured by the suitable combination of docking and Molecular Dynamics.

The most successful predictor is a learned supervised model built on features describing the protein–ligand interaction across the whole set of representative structures from the conformational panel. Attempts to use only some features or some structures leads to poorer performance. This is consistent with the current understanding that functional activation by allosteric ligands is often mediated by the ligand “selecting” some of the conformational states. Information on both selected and nonselected states is required to identify effective binding. This also suggests that the model has learned the relationship between selective binding patterns and functional effect. Therefore, the need for more sophisticated unsupervised algorithm is explained: this relationship is multivariate, not known in its analytical form and complex.

We propose that the ML strategy we have presented here, while demonstrated on a specific but highly challenging case, is not system-specific and could be extended to the study of other allosterically regulated systems: in this context, we propose our method as a valid complement to the selection of allosteric leads for potential drug-development.

## Molecular Dynamics Simulation and Analysis

The protein structure coordinates (PDB ID: 2CG9) for yeast Hsp90 were downloaded from the Protein Databank. Initial poses for ligand docking were derived from previously published models.^20,21,49,50^ MD simulations were run with Gromacs 2018.2^51^ with the Amber03 force field.^52^ The protein–ligand complex was solvated with TIP3P water model in a dodecahedral box with minimal distance from the solute of 1.4 nm, and counterions were added to neutralize the system. After a minimization, the molecules were equilibrated for 100 ps in the NVT ensemble and successively in the NPT ensemble for 100 ps. The simulations were conducted at a constant temperature of 300 K and at a constant pressure of 1 bar, with a coupling time of 2 ps. The electrostatic term was described by using the particle mesh Ewald algorithm,^53^ and the LINCS algorithm^54−56^ was used to constrain all bond lengths. Available ATP parameters for the Amber force field^57^ were used, and ligands topologies were generated using AnteChamber from the AmberTools module of AMBER18^52^ suite. The atomic point charges were generated with the AM1-BCC charge model, and bonded and nonbonded parameters were automatically assigned with the combination rules defined by the AnteChamber module of the Amber Suite. For each ligand-protein complex, a 400 ns of simulation was run. Cluster analysis was performed on a combined metatrajectory of all simulations with the representative ligands. Rigid roto-translation fitting and RMSD calculations were made on α carbon atoms of secondary structure segments extracted with VMD software.^58^ Clustering was performed with Gromos algorithm^59^ using a cutoff between 2 and 2.5 Å.

## Molecular Docking and Fingerprint Analysis

All systems were prepared using the Maestro Software Suite from Schrodinger (www.schrodinger.com): Bond orders and atomic charges were assigned, and the hydrogens were added. Protonation states were evaluated on acid and basic enzymes, and hydrogen bonds were optimized. The protein was then minimized with a Cutoff of 0.3 with respect to starting configuration. The Glide^60^ software was used for molecular docking: the putative binding site was mapped on a grid with dimensions of 48 A, enclosing box, and 28 A, inner box. Calculations with a fixed receptor and flexible ligand were made with standard precision (SP) modality with OPLS3e Force Field. No additional changes to default settings were made. Fingerprint similarities were computed with the Canvas program of the Schrodinger Suite, and the typing scheme is atom distinguished by functional type with no scaling in 32 bit.

## Supervised and Unsupervised Learning

In-house scripts for cluster analysis and supervised learning prediction were developed in Python using scikit-learn functions.^61^ Source code is released under GNU General Public License and available at https://github.com/alepandini/LIGXF. The Logistic Regression models were trained using default settings in scikit-learn. These included L2 norm for penalty estimation with 1e-4 tolerance for stopping criteria and Limited-memory Broyden–Fletcher–Goldfarb–Shanno algorithm (LM-BFGS) for optimization. The SVM models were trained using a linear kernel and all other settings were set to default values. The RF models were trained with an increased number of trees (1000) compared with default, and the best split at decision points was selected by minimization of Gini impurity.
