# Supplementary material for: Machine Learning Prediction of Allosteric Drug Activity from Molecular Dynamics
Source: J Phys Chem Lett. 2021 Apr 12;12(15):3724–32. doi: 10.1021/acs.jpclett.1c00045 (PMC8154828; doi:10.1021/acs.jpclett.1c00045)

Supporting Information for

Machine Learning Prediction of Allosteric Drug Activity from Molecular Dynamics.

Filippo Marchetti[1,2], Elisabetta Moroni[3], Alessandro Pandini[4] e Giorgio Colombo[1,3]

1) Università degli studi di Pavia, Department of Chemistry viale Taramelli 12, Pavia, Italy

2) Università degli studi di Milano, Via C. Golgi, 19, I-20133 Milan, Italy

3) Istituto di Scienze e Tecnologie Chimiche, Via Mario Bianco 9, 20131 Milano, Italy

4) Brunel University London, Uxbridge UB8 3PH, U.K

Supplementary Table S1

The table reports the comparison of the best result obtained by ECFP fingerprint on two clusters with the best ML predictive model obtained by SVM. For SVM the compounds that are correctly predicted are labelled as True, for ECFP we reported the cluster number in which each compound is assigned, the cluster with the majority of inhibitors is 1.

ID	Group	Class	SVM	ECFP
1	Z	activator	False	1
2	BP2	inhibitor	True	1
3	BP2	inhibitor	True	1
4	BP2	inhibitor	True	1
5	BP2	inhibitor	True	1
6	BP2	inhibitor	True	1
7	BP2	inhibitor	False	1
8	BP2	inhibitor	True	1
9	BP2	inhibitor	True	1
10	BP1	inhibitor	True	1
11	BP1	inhibitor	True	1
12	BP1	inhibitor	True	1
13	BP1	inhibitor	True	1
14	BP1	inhibitor	True	1
15	BP2	inhibitor	True	1
16	BP2	inhibitor	True	1
17	BP2	inhibitor	True	1
18	BP2	inhibitor	True	1
19	BP2	inhibitor	True	1
20	BP2	inhibitor	True	1
21	BP2	inhibitor	True	1
22	BP2	inhibitor	True	1
23	BP2	inhibitor	True	1
24	BP2	inhibitor	True	1
25	BP2	inhibitor	True	1
26	BP2	inhibitor	False	1
27	BP2	inhibitor	True	1
28	BP2	inhibitor	False	1
29	BP2	inhibitor	True	1
30	BP2	inhibitor	True	1
31	BP2	inhibitor	True	1
32	BP2	inhibitor	True	1
33	BP2	inhibitor	False	1
34	BP2	inhibitor	True	1
35	BP2	inhibitor	True	1
36	BP2	inhibitor	True	1
37	BP2	inhibitor	True	1
38	BP2	inhibitor	True	1
39	BP2	inhibitor	True	1

40	BP2	inhibitor	True	1
41	BP2	inhibitor	True	1
42	BP2	inhibitor	True	1
43	BP2	inhibitor	True	1
44	BP2	inhibitor	True	1
45	BP2	inhibitor	True	1
46	BP2	inhibitor	True	1
47	BP2	inhibitor	True	1
48	BP2	inhibitor	True	1
49	Ukn	activator	True	1
50	CC	activator	True	2
51	CC	activator	True	2
52	CC	activator	True	2
53	CC	activator	True	2
54	CC	activator	True	2
55	CC	activator	True	2
56	CC	activator	True	2
57	CC	activator	True	2
58	CC	activator	True	2
59	CC	activator	True	2
60	CC	activator	True	2
61	CC	activator	True	2
62	CC	activator	True	2
63	CC	activator	True	2
64	CC	activator	True	2
65	CC	activator	True	2
66	CC	activator	True	2
67	CC	activator	True	2
68	CC	activator	True	2
69	CC	activator	True	2
70	CC	activator	True	1
71	CC	activator	True	2
72	CC	activator	True	2
73	CC	activator	True	2
74	CC	activator	True	2
75	CC	activator	True	2
76	CC	activator	True	2
77	CC	activator	True	2
78	CC	activator	True	2
79	CC	activator	True	2
80	CC	activator	True	2
81	CC	activator	True	2
82	CC	activator	True	2
83	CC	activator	True	2
84	CC	activator	True	2
85	CC	activator	True	2
86	CC	activator	True	2

87	CC	activator	True	2
88	CC	activator	True	2
89	CC	activator	True	2
90	CC	activator	False	2
91	CC	activator	True	2
92	CC	activator	True	2
93	CC	activator	True	2
94	CC	activator	True	2
95	CC	activator	True	2
96	CC	activator	True	2
97	CC	activator	True	2
98	CC	activator	True	2
99	CC	activator	True	2
100	CC	activator	True	2
101	CC	activator	True	2
102	CC	activator	True	2
103	CC	activator	True	2
104	CC	activator	True	2
105	CC	activator	True	2
106	CC	activator	True	2
107	CC	activator	True	2
108	CC	activator	True	2
109	CC	activator	True	2
110	CC	activator	True	2
111	CC	activator	True	2
112	CC	activator	True	2
113	CC	activator	True	2
114	CC	activator	True	2
115	CC	activator	True	2
116	CC	activator	True	2
117	CC	activator	False	2
118	CC	activator	True	2
119	CC	activator	True	2
120	CC	activator	True	2
121	CC	activator	True	2
122	CB	inhibitor	False	1
123	CB	inhibitor	False	1
124	DP	activator	True	1
125	DP	activator	True	1
126	DP	activator	False	1
127	DP	activator	True	1
128	DP	activator	False	1
129	DP	activator	True	1
130	Z	activator	True	1
131	Z	activator	False	1
132	GT	activator	True	1
133	CC	activator	True	2

Supplementary Figure S1

List and molecular structures of inhibitors and activators.

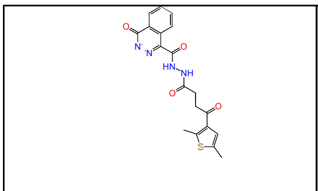<br>entry id: 1	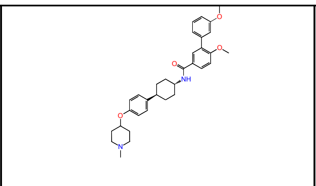<br>entry id: 2	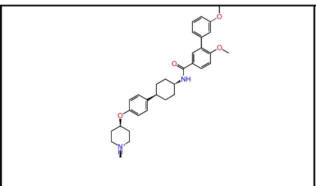<br>entry id: 3	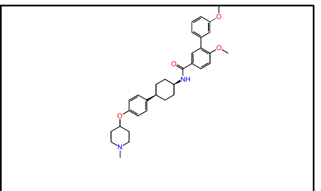<br>entry id: 4
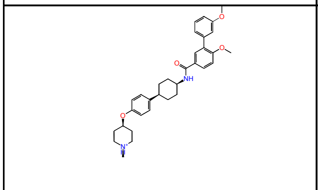<br>entry id: 5	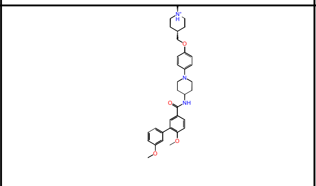<br>entry id: 6	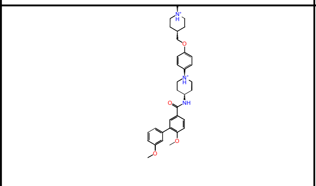<br>entry id: 7	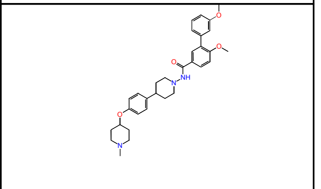<br>entry id: 8
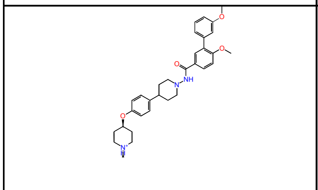<br>entry id: 9	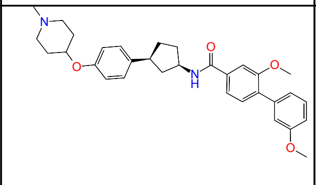<br>entry id: 10	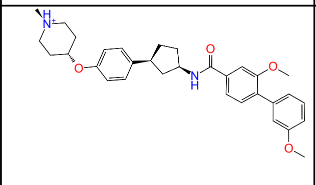<br>entry id: 11	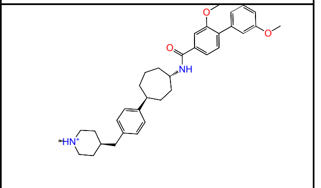<br>entry id: 12
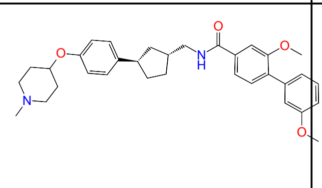<br>entry id: 13	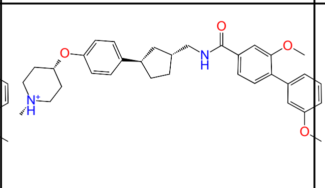<br>entry id: 14	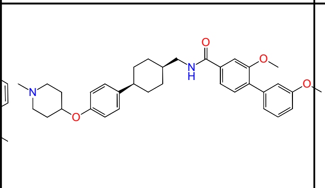<br>entry id: 15	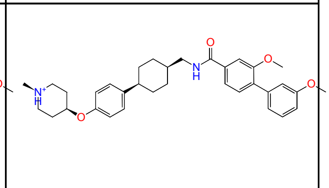<br>entry id: 16
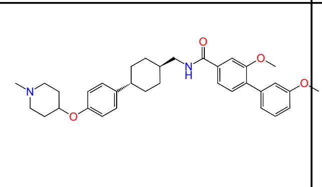<br>entry id: 17	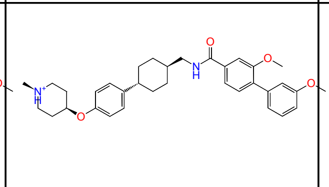<br>entry id: 18	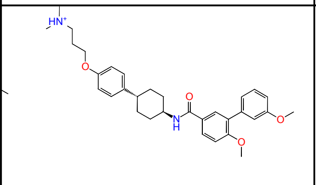<br>entry id: 19	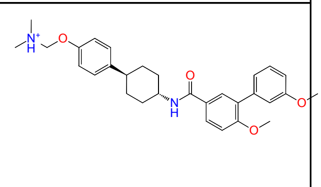<br>entry id: 20
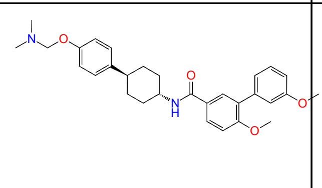<br>entry id: 21	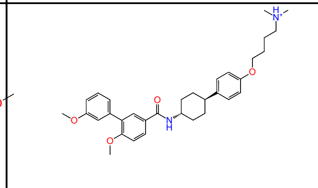<br>entry id: 22	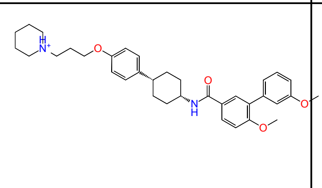<br>entry id: 23	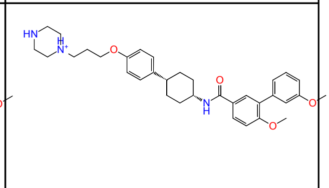<br>entry id: 24
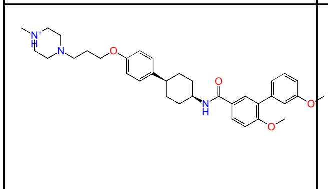<br>entry id: 25	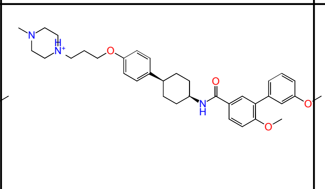<br>entry id: 26	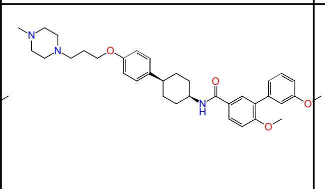<br>entry id: 27	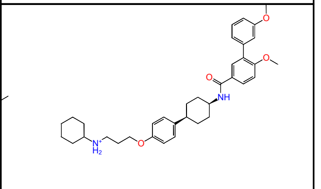<br>entry id: 28
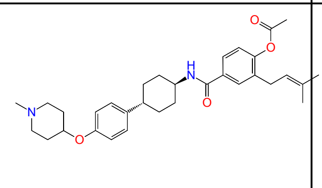<br>entry id: 29	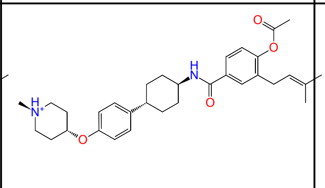<br>entry id: 30	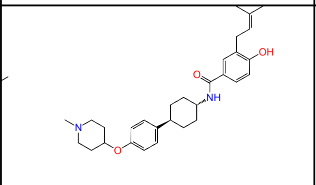<br>entry id: 31	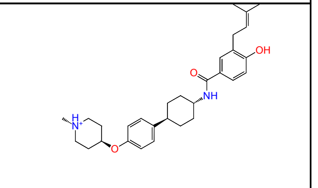<br>entry id: 32

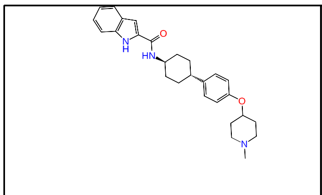
entry id: 33

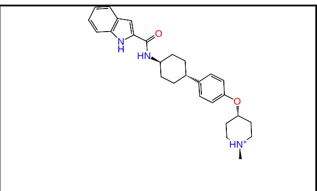
entry id: 34

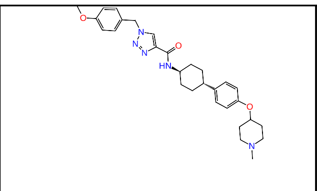
entry id: 35

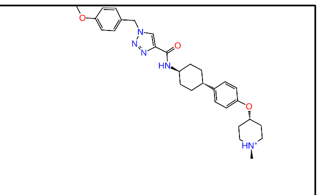
entry id: 36

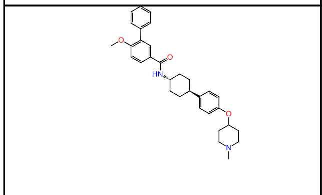
entry id: 37

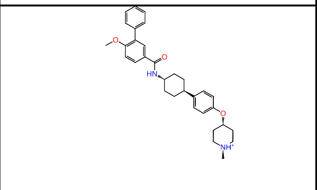
entry id: 38

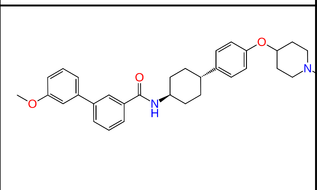
entry id: 39

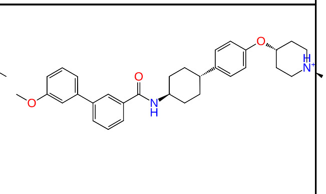
entry id: 40

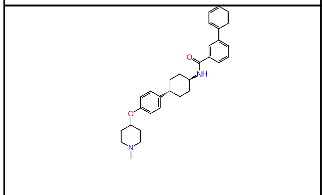
entry id: 41

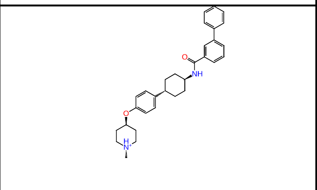
entry id: 42

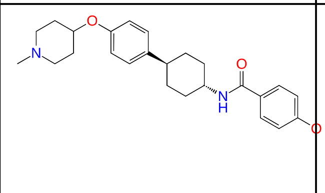
entry id: 43

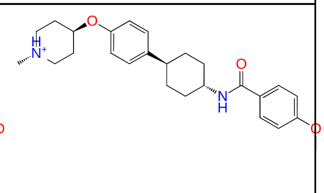
entry id: 44

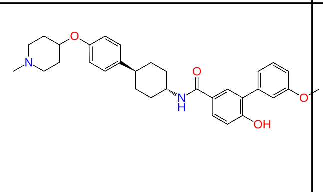
entry id: 45

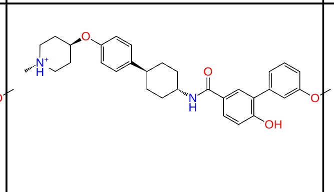
entry id: 46

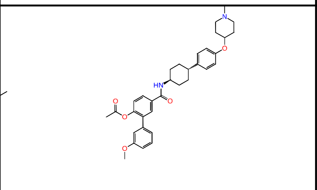
entry id: 47

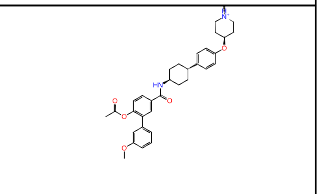
entry id: 48

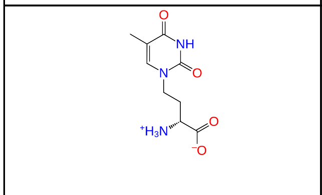
entry id: 49

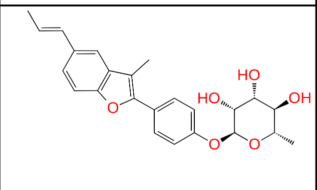
entry id: 50

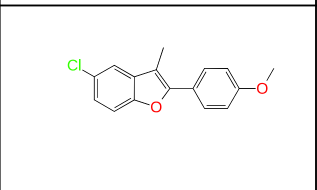
entry id: 51

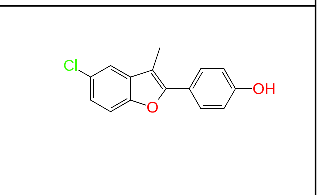
entry id: 52

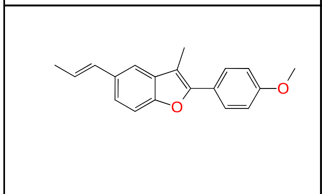
entry id: 53

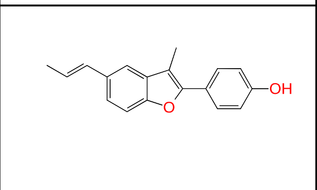
entry id: 54

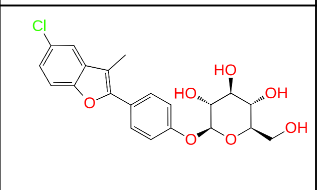
entry id: 55

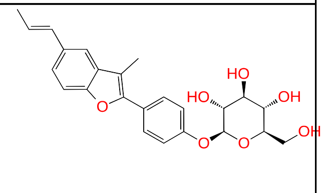
entry id: 56

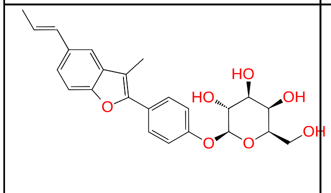
entry id: 57

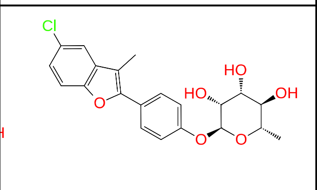
entry id: 58

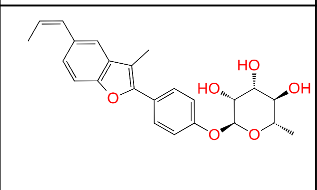
entry id: 59

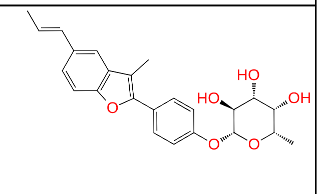
entry id: 60

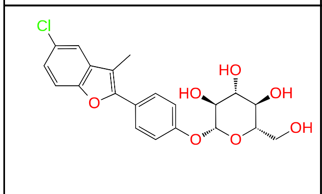
entry id: 61

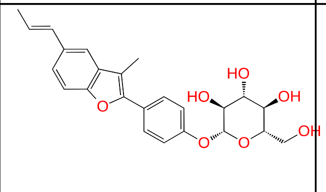
entry id: 62

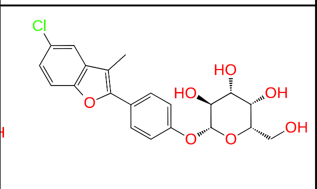
entry id: 63

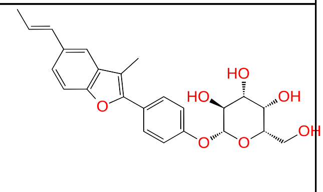
entry id: 64

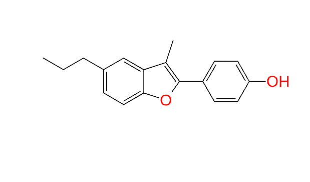

entry id: 65

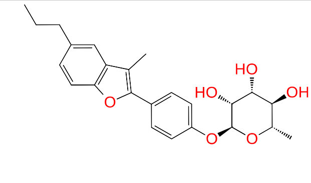

entry id: 66

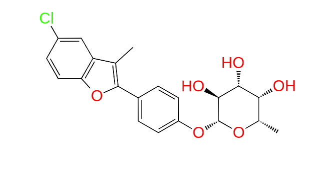

entry id: 67

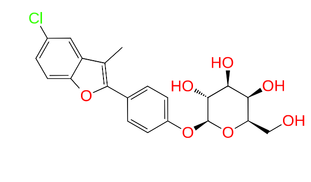

entry id: 68

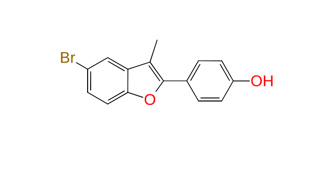

entry id: 69

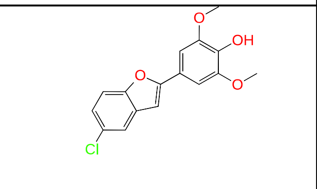

entry id: 70

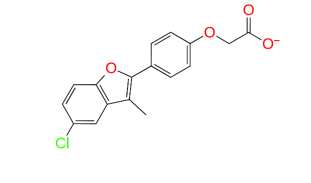

entry id: 71

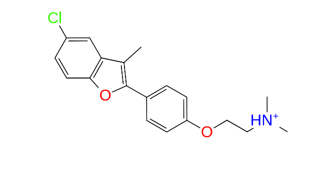

entry id: 72

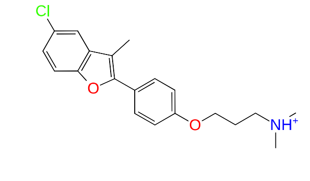

entry id: 73

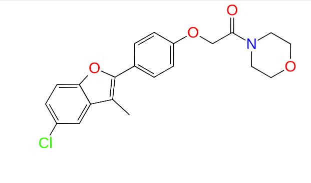

entry id: 74

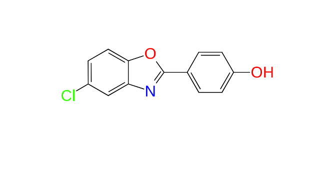

entry id: 75

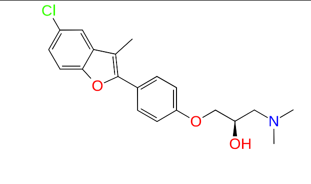

entry id: 76

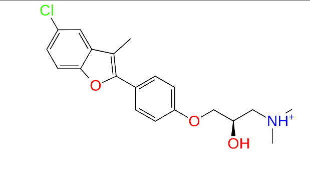

entry id: 77

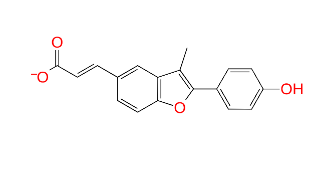

entry id: 78

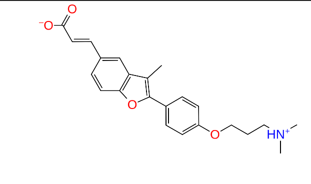

entry id: 79

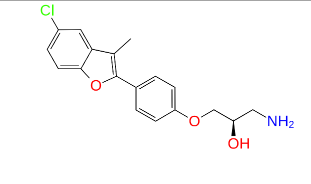

entry id: 80

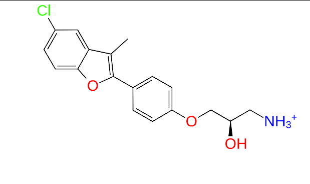

entry id: 81

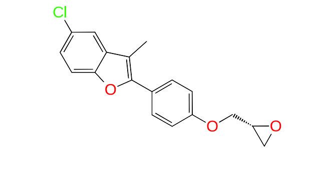

entry id: 82

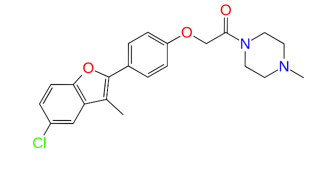

entry id: 83

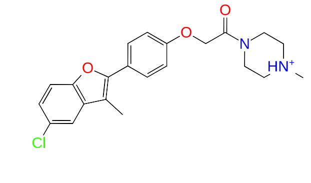

entry id: 84

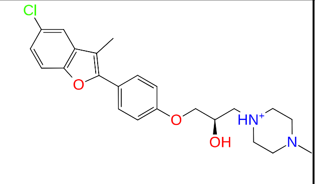

entry id: 85

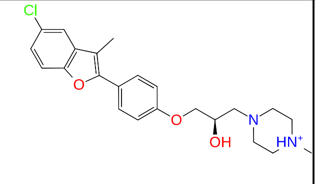

entry id: 86

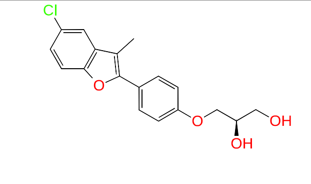

entry id: 87

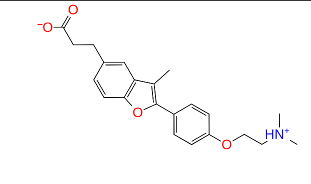

entry id: 88

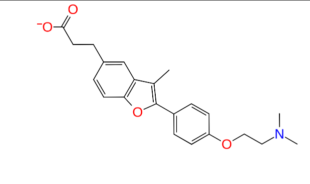

entry id: 89

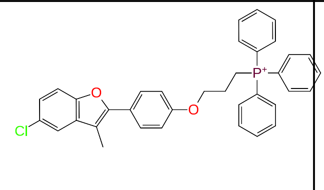

entry id: 90

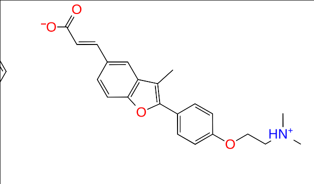

entry id: 91

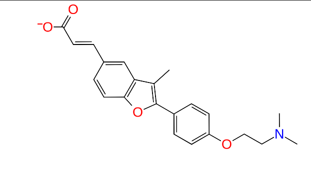

entry id: 92

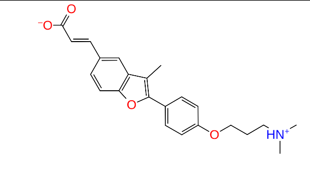

entry id: 93

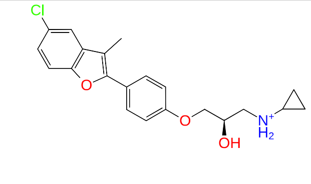

entry id: 94

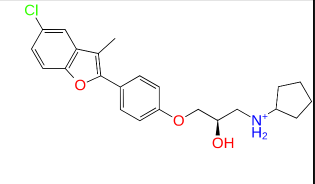

entry id: 95

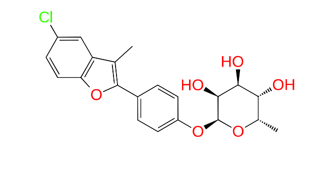

entry id: 96

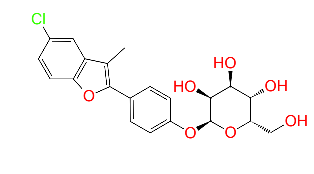
entry id: 97

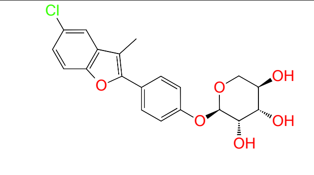
entry id: 98

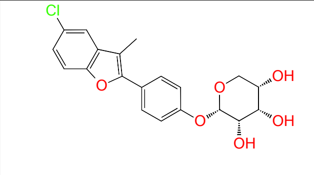
entry id: 99

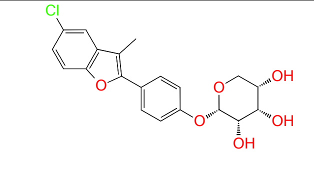
entry id: 100

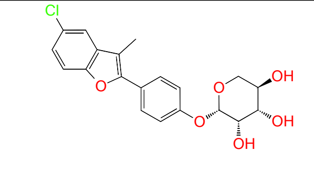
entry id: 101

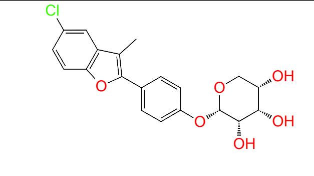
entry id: 102

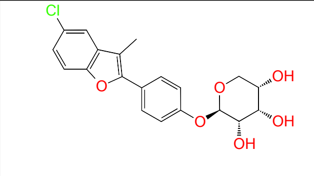
entry id: 103

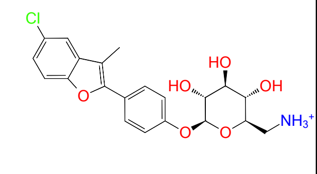
entry id: 104

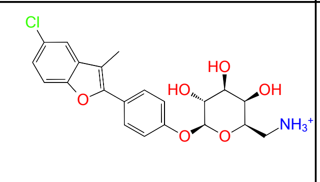
entry id: 105

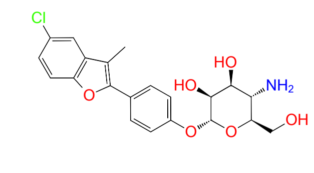
entry id: 106

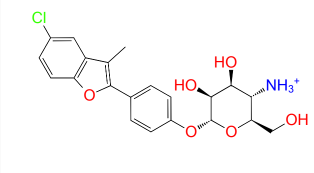
entry id: 107

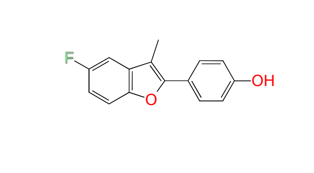
entry id: 108

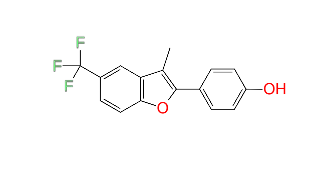
entry id: 109

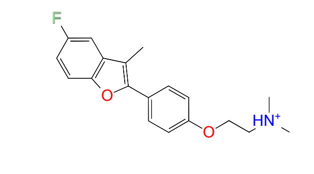
entry id: 110

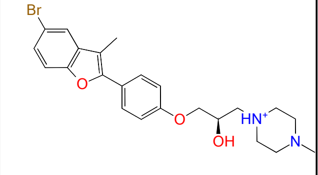
entry id: 111

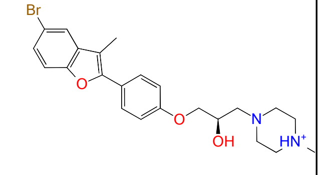
entry id: 112

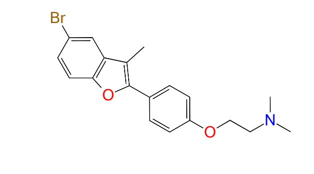
entry id: 113

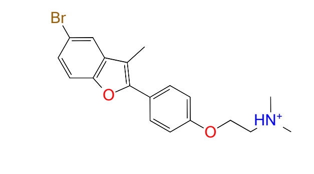
entry id: 114

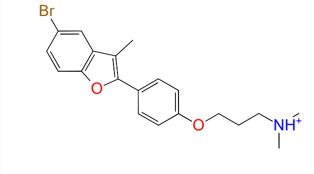
entry id: 115

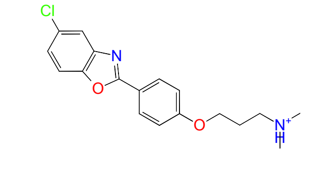
entry id: 116

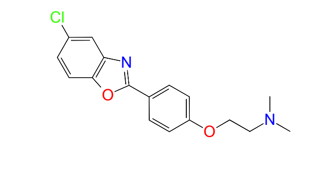
entry id: 117

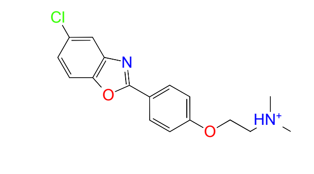
entry id: 118

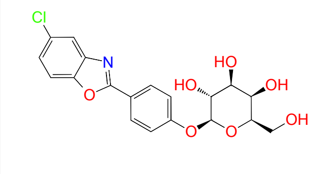
entry id: 119

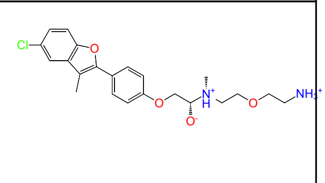
entry id: 120

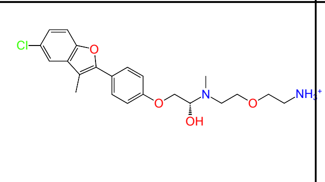
entry id: 121

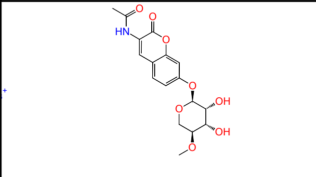
entry id: 122

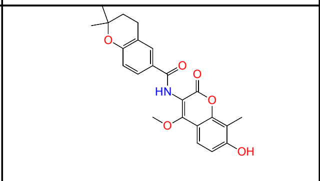
entry id: 123

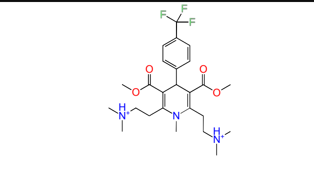
entry id: 124

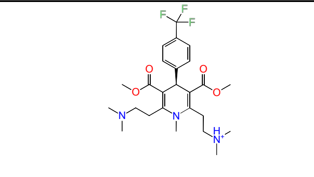
entry id: 125

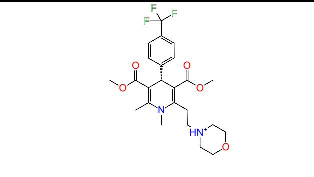
entry id: 126

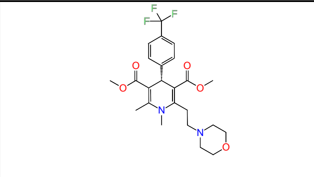
entry id: 127

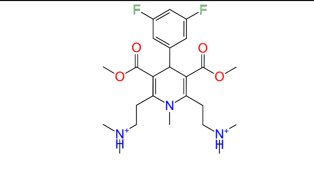
entry id: 128

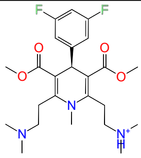

entry id: 129

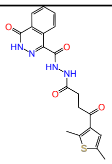

entry id: 130

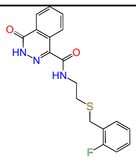

entry id: 131

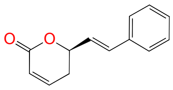

entry id: 132

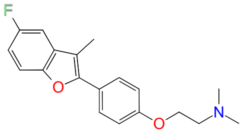

entry id: 133

Supplementary Figure S2

The ligands forming complexes with Hsp90 and used in the MD simulations.

CheCOSP26

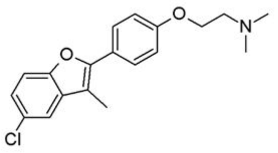

ND2

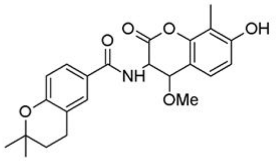

Novobiocin

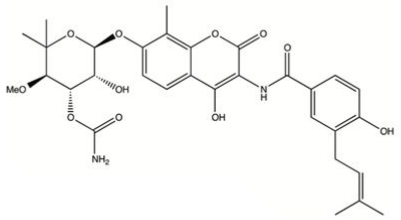

Supplementary Figure S3

Here are reported the boxplots of the distribution of values for every single feature against the two known ligand classes: inhibitors and activators. The legend of the features is:

Score= docking score for the best pose.

RMSD= rmsd of the docking score for the ten best poses.

RMS= RMS of atomic position respect to the best pose, averaged over the ten best poses.

Bs= Clustering on the binding site.

Nd= Clustering on the N-term domain.

Md= Clustering on the Middle domain.

NM= Clustering on both N-term and Middle domains.

MC= Clustering on both Middle and C-term domain.

Numbers 1,2,3= First, Second and Third representative structure of the cluster analysis.

Inhibitors are in red and activators are in orange.

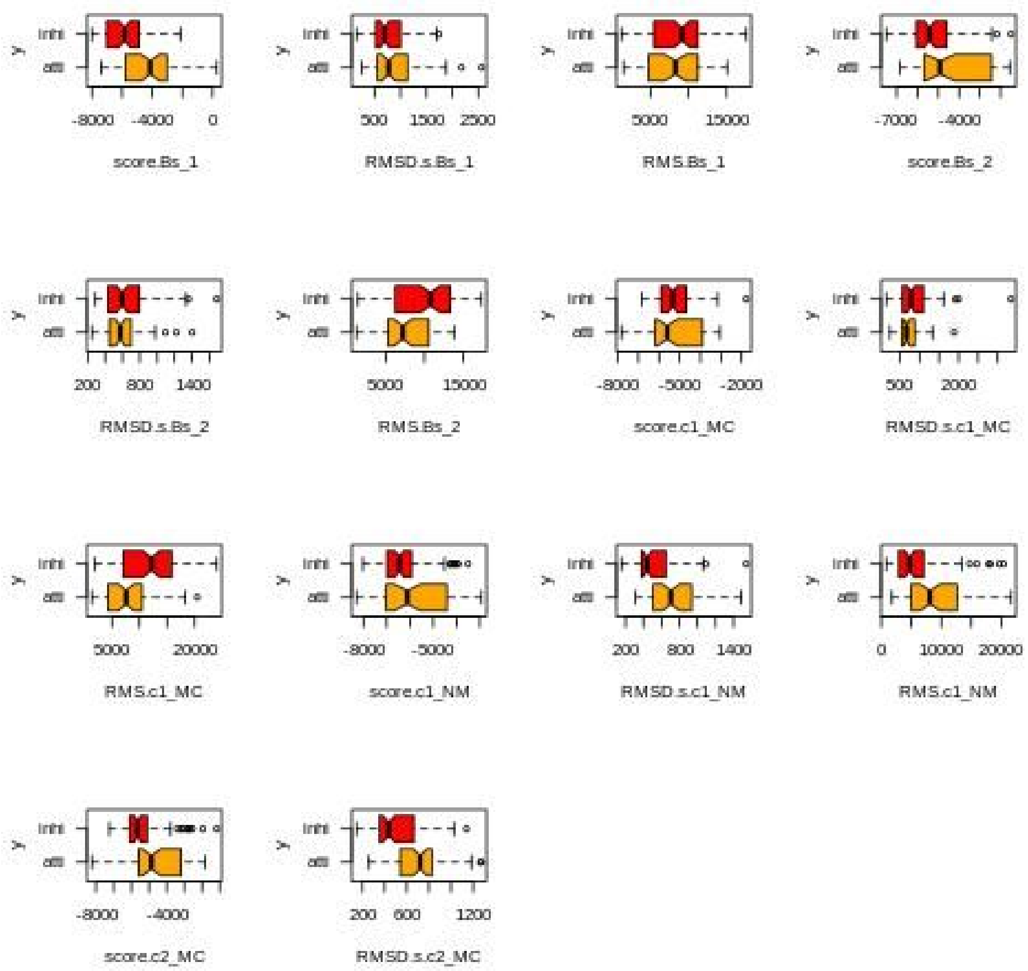

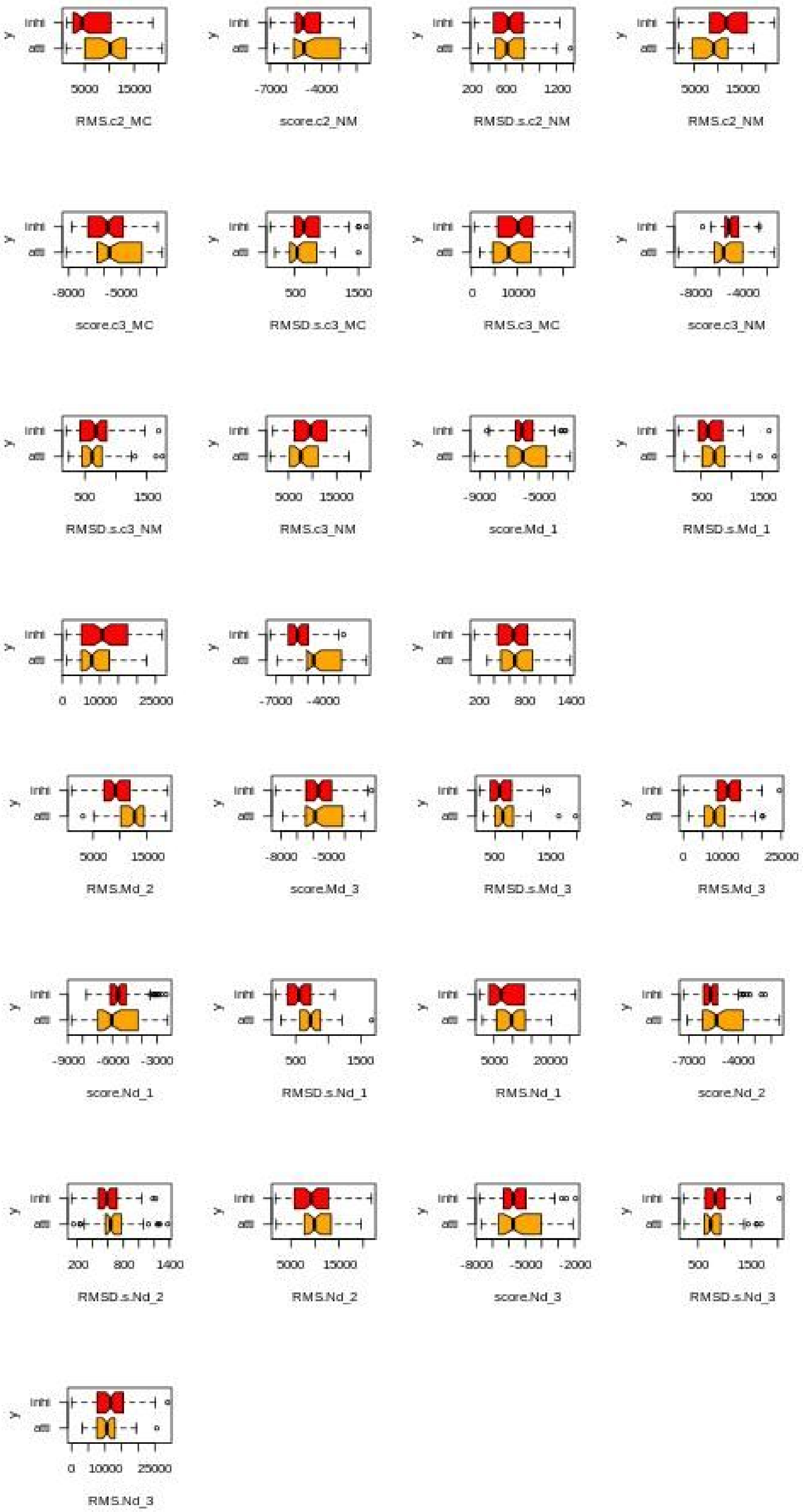

5000 15000
RMS.c2_MC
-7000 -4000
score.c2_NM
200 600 1200
RMSD.s.c2_NM
5000 15000
RMS.c2_NM
-8000 -5000
score.c3_MC
500 1500
RMSD.s.c3_MC
0 10000
RMS.c3_MC
-8000 -4000
score.c3_NM
500 1500
RMSD.s.c3_NM
5000 15000
RMS.c3_NM
-9000 -5000
score.Md_1
500 1500
RMSD.s.Md_1
0 10000 25000
-7000 -4000
200 800 1400
5000 15000
RMS.Md_2
-8000 -5000
score.Md_3
500 1500
RMSD.s.Md_3
0 10000 25000
RMS.Md_3
-9000 -6000 -3000
score.Nd_1
500 1500
RMSD.s.Nd_1
5000 20000
RMS.Nd_1
-7000 -4000
score.Nd_2
200 800 1400
RMSD.s.Nd_2
5000 15000
RMS.Nd_2
-8000 -5000 -2000
score.Nd_3
500 1500
RMSD.s.Nd_3
0 10000 25000
RMS.Nd_3

Supplementary Figure S4

The distribution of balanced accuracy for SVM models is reported for different subset size of the original dataset. For each subset size training and test were repeated on 100 random samples.

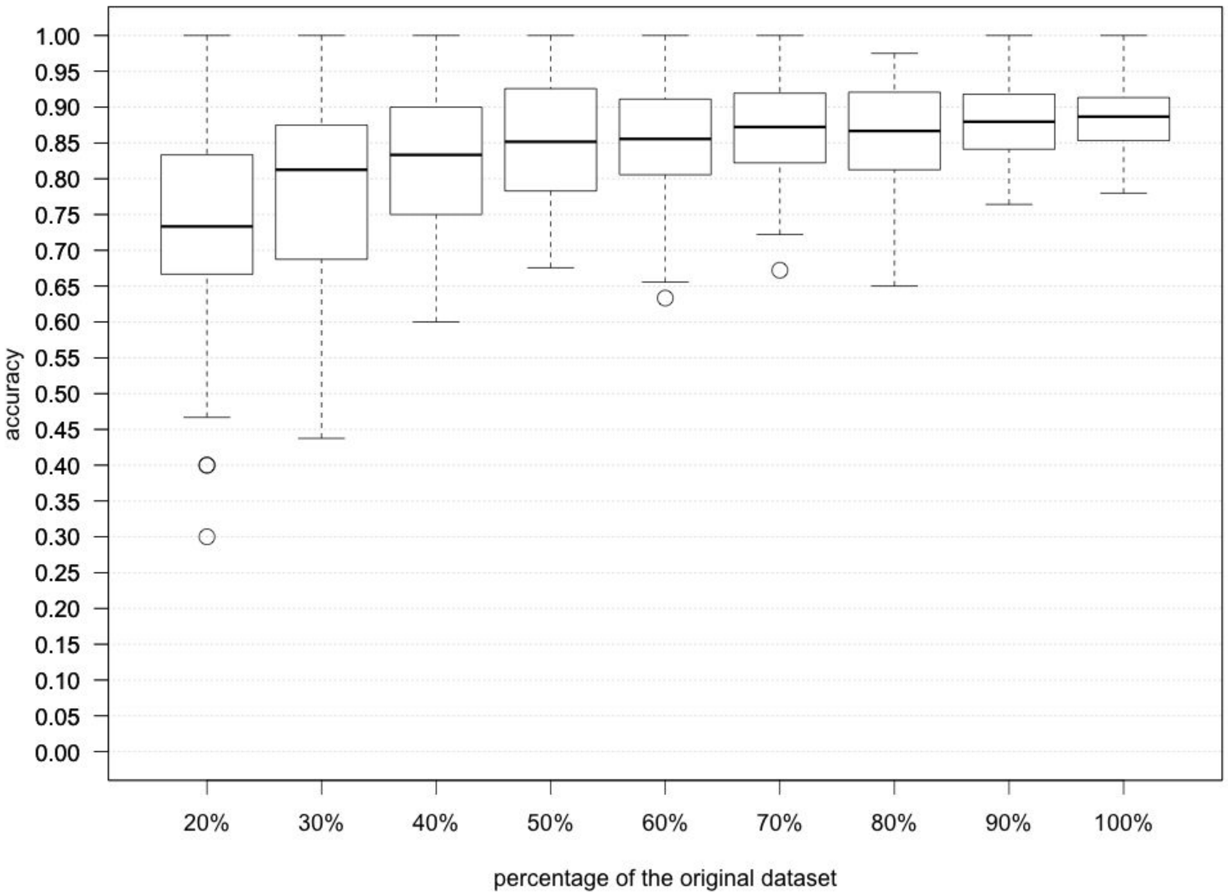

Supplementary Figure S5

Boxplot of accuracy distribution after a cross-validation cycle, comparison of SVM model trained with all the features with the SVM trained after the drop-out. Every sample represents a crossvalidation cycle.

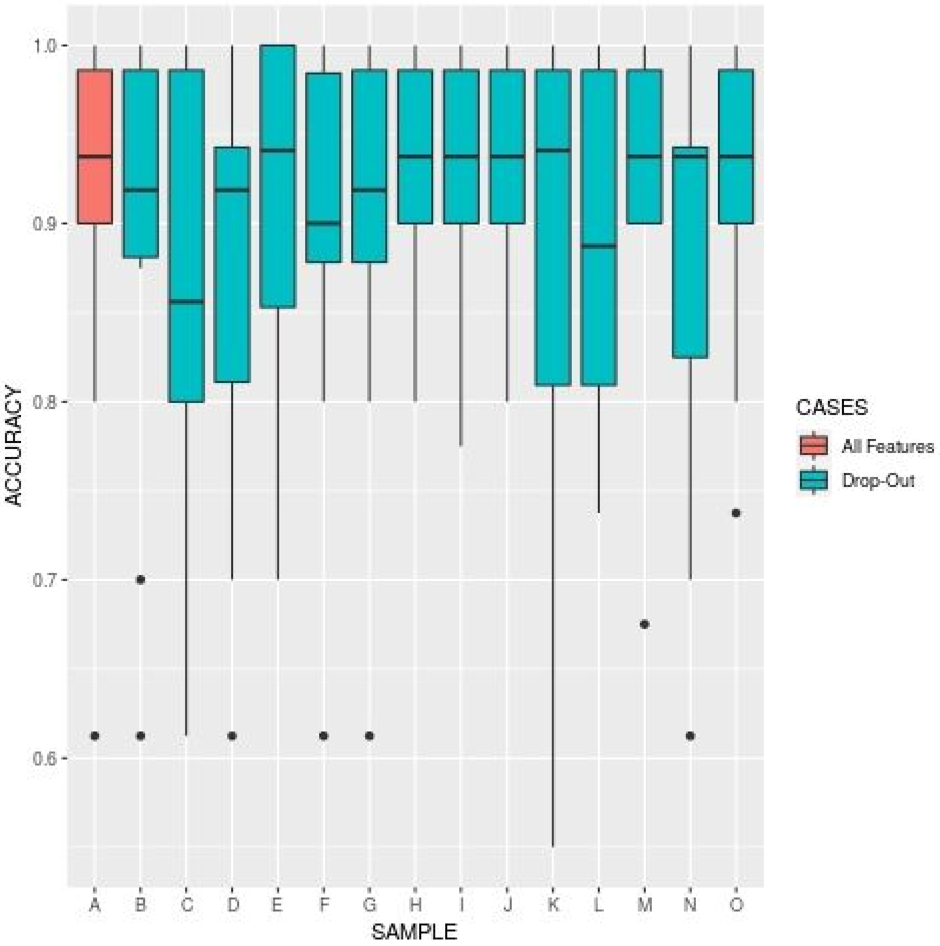

Supplement: Supplementary file 1 — jz1c00045_si_001.pdf [file jz1c00045_si_001.pdf]
